# Optimum Accuracy of Massive Transfusion Protocol Activation: The Clinician’s View

**DOI:** 10.7759/cureus.3688

**Published:** 2018-12-05

**Authors:** Chris Bell, Oksana Prokopchuk-Gauk, Bruce Cload, Alena Stirling, Philip J Davis

**Affiliations:** 1 Internal Medicine, University of Saskatchewan College of Medicine, Saskatoon, CAN; 2 Pathology and Laboratory Medicine, University of Saskatchewan, Royal University Hospital, Saskatoon, CAN; 3 Emergency Medicine, University of Saskatchewan, Royal University Hospital, Saskatoon, CAN; 4 Anaesthesia, University of Saskatchewan, Royal University Hospital, Saskatoon, CAN

**Keywords:** massive tranfusion protocol, expert opinion, accuracy, over-activation rate, under-activation rate

## Abstract

Background

Massive transfusion protocols (MTP) aid in the efficient delivery of blood components to rapidly exsanguinating patients. Unfortunately, clinical gestalt and currently available clinical scoring systems lack the optimal accuracy to prevent blood product wastage (through over-activation), as well as individual patient morbidity and mortality (through under-activation). In order to help refine the MTP activation criteria and protocols, we surveyed clinicians on acceptable over- and under-activation rates for massive transfusions.

Methods

We surveyed Canadian content experts in their respective fields, using a snowball survey technique. Respondents were categorized into two groups: Group 1 was comprised of trauma and acute care specialists (TACS), while Group 2 was comprised of clinical and laboratory medicine specialists (CLMS). Between-group differences were examined using Fisher’s exact test and the likelihood ratio. Statistical significance was set at p < 0.05.

Results

We received responses from 35 clinicians in the TACS group and 10 clinicians in the CLMS group. About half (45.7%) of respondents in the TACS group considered an MTP overactivation rate of 5% - 10% acceptable (vs. 60% of the CLMS group; not significant (NS)). Approximately one-third (34.2%) of the respondents in the TACS group considered an MTP under-activation rate of less than 5% acceptable, whereas the majority (60%) of respondents in the CLMS group considered an under-activation rate of less than 5% acceptable (NS). A significantly greater proportion of respondents in the TACS group felt that an anticipated need for > 20 units of packed red blood cells within the next 24 hours was an acceptable criterion for MTP activation. Respondents in the CLMS group were more likely to consider “poor communication” as a reason for blood component wastage.

Conclusion

Similarities in acceptable MTP over- and under-activation rates were noted across specialties. Collaboration between involved parties is necessary for MTP protocol development to improve patient outcomes and reduce blood wastage.

## Introduction

While various definitions of massive transfusion exist, massive transfusion is most commonly defined as the transfusion of ≥ 10 units of packed red blood cells (pRBCs) in 24 hours [1]. Massive transfusion protocols (MTP) allow for the efficient delivery of large quantities of blood components to the rapidly exsanguinating patient [1-3]. Despite being somewhat algorithmic, MTPs are required in complex medical scenarios, and the considerations for MTP activation are often constrained by limited time and information [3-4]. 

An effective way to optimize patient outcomes is carefully selected and adhered to MTP activation criteria [5]. Critical Care Canada Guidelines advocate that these criteria are established in consultation with all involved medical specialties and with a comprehensive institutional plan to maximize patient outcomes, deliver a reasonable ratio of blood components, and minimize unnecessary wastage of blood components [6]. Unfortunately, established protocols and MTP activation criteria still require a fair amount of clinical gestalt which, at times, can be biased and potentially result in over-triage where patients who do not require activation of an MTP receive blood products through activation of an MTP [7-9]. This can cause harm as MTP "under-activation" places undue risk upon the individual patient, while "over-activation" can result in the overuse and/or wastage of blood components. That said, when needed, “every minute” of delay in receiving blood components increases patient mortality [4]. Further, currently available scoring systems, such as the "Assessment of Blood Consumption" (ABC) Score and "Shock Index", are lacking in varying degrees of sensitivity and specificity [9-10]. Therefore, clarifying clinically acceptable rates of over- and under-activation may sharpen MTP activation criteria and protocols, as well as set benchmarks for trauma and acute care systems. 

To our knowledge, no study has examined the clinician's perspective on appropriate levels of over- and under-activation for massive transfusions. Thus, the purpose of this study was to survey physicians currently practicing in Canada commonly involved in MTP activation and administration to determine their perceptions of "ideal" MTP over- and under-activation rates.

## Materials and methods

Survey development and distribution

In consultation with the Social Sciences Research Laboratory (SSRL) at the University of Saskatchewan, we developed a 20-question survey to assess respondent demographics, opinions regarding MTP protocols, perceptions of appropriate MTP activation criteria, and acceptable rates of over- and under-activation. Respondents were also questioned about whether or not they would activate an MTP using case-based scenarios. A copy of the survey appears in Appendix 1.

We had initially planned to distribute the survey through Canadian specialist associations; however, not all respective associations had a survey mechanism in place. As such, we elected to distribute the survey to peer-identified content experts in the fields of Emergency Medicine, Anesthesia, Critical Care, General and Trauma Surgery, Hematology, Hematopathology, and Transfusion Medicine using a “snowball” survey technique. Initial survey respondents were content experts known to the study authors. These initial respondents subsequently provided names and contact information for additional Canadian content experts, ultimately "snowballing" into a growing list of participants. The survey was primarily distributed using the online survey platform SurveyMonkey® (http://www.surveymonkey.com). Prior to the survey, potential respondents were supplied with a participant information form that outlined the purpose of the study and the risks and benefits of participation. Responding to the survey implied consent. The survey was password protected for participant security, privacy, and anonymity and was accessible for six weeks. The University of Saskatchewan Research Ethics Board approved this study (REB #17-166).

Statistical analysis

For comparison purposes, physician respondents were categorized into one of two groups: Group 1 was comprised of trauma and acute care specialists (TACS) practicing in trauma care, emergency medicine, anesthesia, critical care, and surgery; Group 2 included clinical and laboratory medicine specialists (CLMS) practicing in hematology, hematopathology, and transfusion medicine. Statistical analysis was performed by the SSRL, and between-group differences were assessed using Fisher’s exact test for 2 x 2 tables and the likelihood ratios for tables larger than 2 x 2. Statistical significance was set at p < 0.05.

## Results

Our survey was distributed to 83 Canadian physicians, and we received a 54.2% response rate (45 respondents), including representative responses from the majority of the Canadian Provinces. Only one respondent worked in a hospital that did not have an MTP. Of the 45 respondents, 35 respondents were in the TACS group, and 10 were in the CLMS group. Just over half (53.3%) of the respondents practiced in an area whose population size was less than 500,000, 24.4% in a population between 500,000 and one million, and 22.2% in a population of more than one million people (Table 1). When the number of years in practice was considered, 17.8% of respondents had been practicing for 0 - 5 years, 35.6% had been practicing for 6 - 10 years, 26.7% had been practicing for 11 - 20 years, 13.3% had been practicing for 21 - 30 years, and 6.7% had been practicing for more than 30 years at the time of completing our survey.

**Table 1:** Respondent Demographics CLMS: clinical and laboratory medicine specialists; N: number; TACS: trauma and acute care specialists

	TACS	CLMS
Population Size (N)		
< 500,0000	22	2
500,000-1,000,000	9	2
> 1,000,000	4	6
Province (N)		
British Columbia	1	2
Alberta	4	1
Saskatchewan	12	2
Manitoba	2	
Ontario	11	3
Quebec		1
New Brunswick		
Nova Scotia	4	1
PEI	1	
Newfoundland		
Years in Practice (N)		
0 - 5	7	1
6 - 10	10	6
11 - 20	9	3
21 - 30	6	0
> 30	3	0
Specialty (N)		
Emergency Medicine	16	
Surgery (General and Vascular)	13	
Anesthesia and Critical Care/Intensive Care	6	
Transfusion Medicine, Hematology and Hematopathology		10

Fifty-percent of respondents in the CLMS group responded that they worked in a hospital whose MTP included an activation criterion for a peripartum woman with marked ongoing blood loss (vs. 8.6% of the TACS group, p < 0.008). In the TACS group, 34.2% of the physicians felt an under-activation rate of less than 5% was acceptable, while the majority (60%) of physicians in the CLMS group felt that an under-activation rate of less than 5% was acceptable (p = 0.120). In the TACS group, 45.7% of physicians felt an over-activation rate of 5% to 10% was acceptable, while the majority (60%) of physicians in the CLMS group felt that an over-activation rate of 5% to 10% was acceptable (p = 0.361).

**Figure 1:**
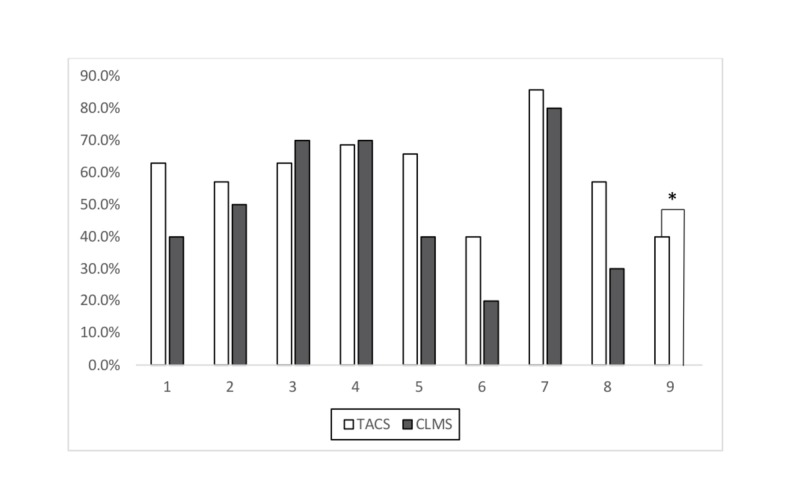
‘Reasonable’ massive transfusion protocols (MTP) activation criteria by specialist group 1: > 4.5 L of blood loss in 30 minutes 2: > 150 mL per minute of bleeding with loss of over half of the circulating blood volume 3: Peripartum woman with marked ongoing blood loss 4: Systolic blood pressure < 90 and/or requiring vasopressors from presumed hemorrhagic shock 5: Replacement of 50% of the total blood volume (TBV) within three hours (h) foreseen 6: Replacement of one entire blood volume within 24 h 7: Transfusion of > 4 units of packed red blood cells (pRBCs) in 1 h when ongoing need is foreseeable 8: Anticipated transfusion of > 10 units of pRBCs in the next 24 h 9: Anticipated transfusion of > 20 units of pRBCs in the next 24 h * = p < 0.05 CLMS: clinical and laboratory medicine specialists; TACS: trauma and acute care specialists

Figure 1 summarizes the differences in perceptions about reasonable MTP activation criteria. While 40% of respondents in the TACS group felt that an “anticipated transfusion of > 20 units of packed red blood cells (pRBCs) in 24 hours" was an appropriate criterion for MTP activation, no respondents in the CLMS group felt that this was an appropriate MTP activation criterion (p = 0.019). 

**Figure 2:**
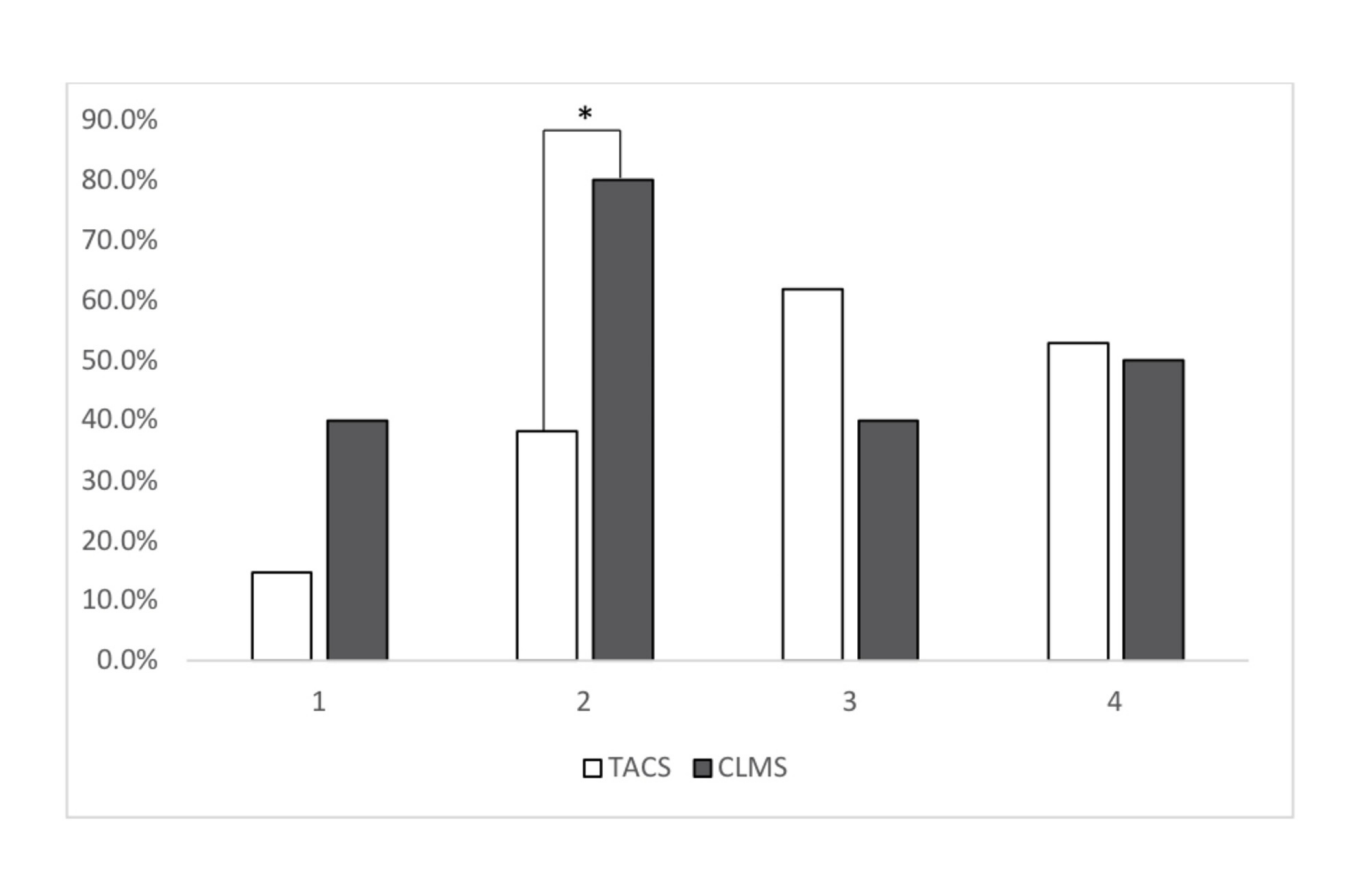
Comparison between specialist groups regarding opinions for perceived blood component wastage 1: Inappropriate activation of a massive transfusion protocol (MTP) 2: Poor communication between treating services 3: Maximum time frame during which blood components can be out of the blood bank before expiry, according to local validated MTP box protocols 4: Over-anticipation of the amount of blood products needed * = p < 0.05 CLMS: clinical and laboratory medicine specialists; TACS: trauma and acute care specialists

Figure 2 summarizes the perceived reasons for blood wastage in MTP activations. In comparing the TACS and CLMS groups, the CLMS group was more likely to feel that poor communication between treating services (80.0% versus 38.2% of the TACS group; p = 0.031) was a reason for blood product wastage.

**Figure 3:**
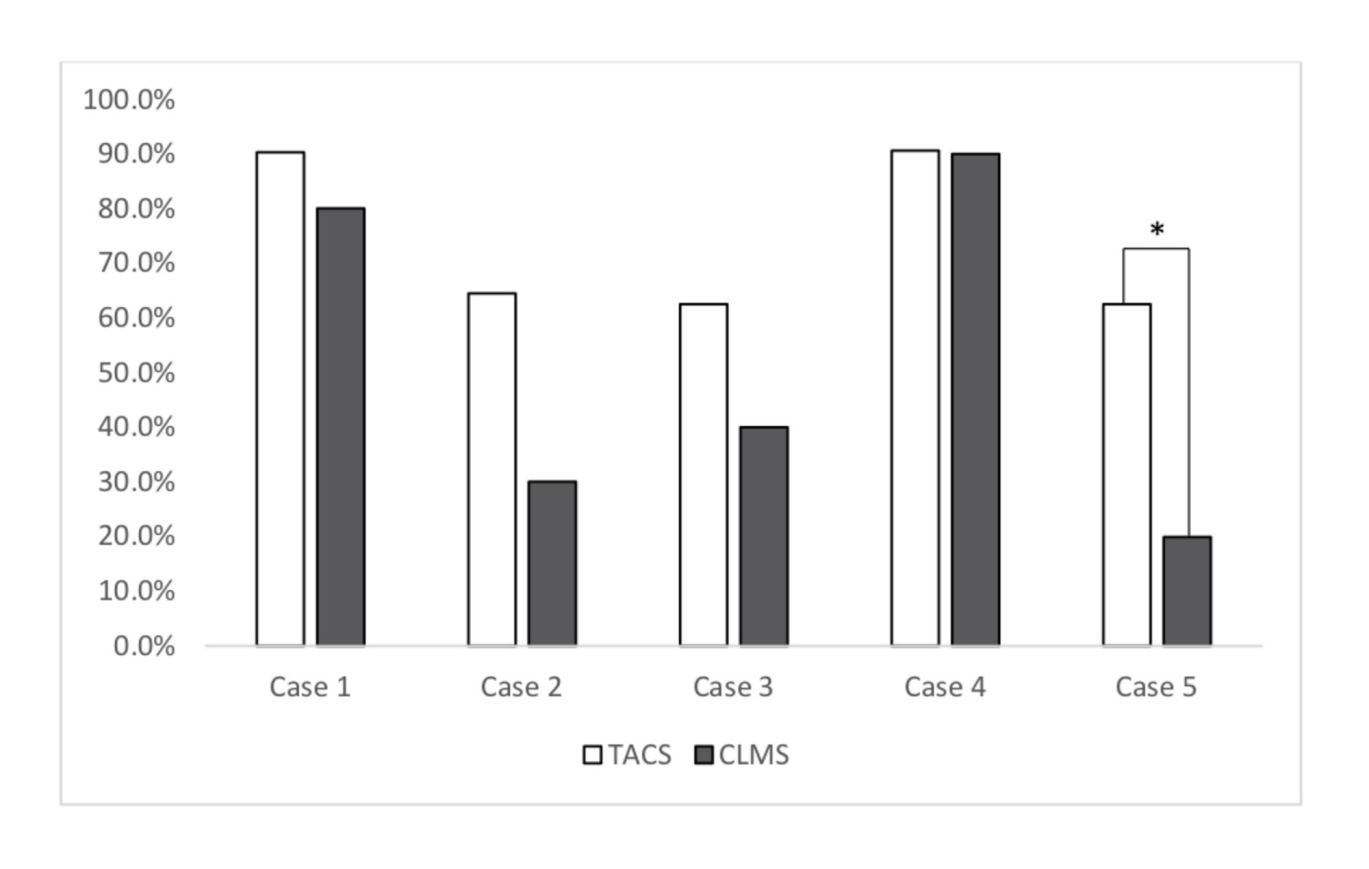
Comparison of TACS and CLMS physician for MTP activation in case-based scenarios Case #1: Pelvic crush Injury Case #2: Motor vehicle accident Case #3: Bleeding abdominal aortic aneurysm Case #4: Third-trimester bleeding Case #5: Gastrointestinal bleeding * = p < 0.05 CLMS: clinical and laboratory medicine specialists; MTP: massive transfusion protocols; TACS: trauma and acute care specialists

A practical assessment of MTP activation decisions was evaluated through survey responses to five clinical scenarios (Figure 3). Case 1 described a pelvic crush injury. Case 2 described an elderly patient with blunt abdominal and pelvic trauma after a motor vehicle collision. Case 3 described a patient with a bleeding postoperative abdominal aortic aneurysm (AAA). Case 4 described a third-trimester bleed from a presumed placental abruption. Case 5 described a massive gastrointestinal (GI) bleed. Key differences arose for the massive GI bleed case where 62.5% of the TACS group would activate an MTP vs. 20% of the CLMS group (p = 0.03).

## Discussion

We examined the clinician’s perspective on acceptable rates of under- and over-activation of MTPs. Interestingly, although not statistically significant, the majority of respondents in the CLMS group appeared to favor a lower under-activation rate for MTP activation. This approach would favor giving blood to the individual patient and suggests a confidence among CLMS in blood bank resources available to support bleeding patients while maintaining an adequate supply for routine use. This approach is consistent with the conclusions of a University of Pittsburgh study which showed that despite an over-activation rate of 53.8% in non-trauma patients, the benefits of MTP activation for an individual patient outweighed the potential detriment to system resources [11]. Given that MTP activation does not appear to be associated with increased waste, blood component management during an MTP appears to be more important than whether or not an MTP was activated. Furthermore, in the trauma population at least, “every minute” appears to count, as each minute of delay in receiving blood appears to be associated with increased patient morbidity and mortality [4, 12-14]. Ironically, a more liberal activation of an MTP may paradoxically reduce blood component wastage, as 25% of severely injured patients are coagulopathic [15], and early aggressive resuscitation may reduce the overall blood need [14]. 

Our results suggest that TACS are more likely to consider an “anticipated transfusion of > 20 units of pRBCs in the next 24 hours” appropriate for an MTP activation than CLMS (Figure 1). This criterion reflects a perceived degree of patient blood loss, rather than a measurable amount of blood loss, and the difference in responses likely reflects different clinical perceptions between bedside versus consultative care and the all too common predicament that the degree of hemorrhagic shock is difficult to predict [16-17]. This is likely why various institutional MTP protocols allow for a fair amount of clinical gestalt, which, on its own, has only moderate sensitivity and specificity but can be combined with an institutional protocol or clinical prediction tool to improve the overall accuracy of an MTP prediction [9, 17].

Despite the best efforts of physicians, MTP activations result in a blood component waste which is a universal concern for all specialties [1, 6]. In our study, both groups agreed that “over-anticipation of blood product need” was a common contributor to blood waste (Figure 2). This is consistent with recent literature showing that over-anticipation of blood need, as well as limitations on safe storage times outside of the blood bank, result in blood component wastage rates of 0 - 9% (for red blood cells) at their lowest and 0 - 33% (for cryoprecipitate) at their highest [18]. About one-third (36.5%) of our respondents practiced in institutions where components could not be returned to the blood bank beyond one hour from the time of issue, suggesting that cooler times for refrigerated products had not been validated to allow for a period of extended component storage. Lastly, CLMS were statistically more likely to consider “poor communication” as a reason for blood product wastage. Poor communication about the patient’s ongoing need for blood components has been shown to occur in up to one-third of cases [6], and difficulties in maintaining dynamic quantities of blood components in close proximity to patients that are moving through multiple hospital locations over a period of time can create potential issues. This suggests that there is room for improving closed loop communication between treating physicians and the lab, which may be enhanced through ongoing education and quality assurance initiatives [19-22]. Solutions to these problems could include regular meetings between treating services, encouraging physicians to collaborate via interprofessional grand rounds, and a multidisciplinary performance improvement group [20-21].  

Finally, we examined differences in approach to MTP activation between the two studied groups of physicians. In our study, physicians in the TACS group was significantly more likely to activate an MTP in only one of the case scenarios, which involved a post-arrest patient found to have a massive GI bleed (Figure 3). Differences in activation rates between the two specialty groups may not be surprising, as TACS may be more likely to rely on clinical gestalt than set activation criteria [16]. Further, MTP activation in non-trauma patients is highly variable, with estimations ranging from 8% - 50% of all MTPs [22]. Given that there is a lack of clinical research that has established objective criteria for activation of an MTP in non-trauma patients, specialists whose primary patient population are not treated in a trauma setting may be at a significant disadvantage, which may explain the disparity in activation between specialist groups. The development of more precise guidelines with regards to activation of MTPs in non-trauma patients would likely benefit both trauma and non-trauma specialists alike.

Limitations

Our study had several limitations. Firstly, our data were collected using a “snowball” survey technique, which has the potential to introduce selection bias, as not all content experts may be invited to participate. Secondly, there is the potential that only content experts with similar views to prior survey respondents may be invited to participate. Lastly, the snowball technique is not random, and responses may not be fully representative of that population. This survey method was chosen for logistical reasons, as we had initially planned to distribute the survey through specialist associations nationally. Unfortunately, not all of these specialist groups have a survey mechanism in place. Furthermore, the specialist practice can be highly varied, and it is conceivable that, depending on practice patterns, the vast majority of respondents surveyed in this manner would not routinely be involved in MTP activation or administration. A final limitation of the study is that, despite a reasonable response rate and low-time commitment, our survey may have been biased by only attracting a relatively small number of respondents who potentially had strong opinions towards MTP activation. This, unfortunately, is inherent in any survey design, and given that there was a minimal disparity in survey responses within groups, we suggest that this is minimal.

## Conclusions

Similarities in acceptable over- and under-activation rates of MTP highlight similar values with respect to MTPs across different specialties. Barriers to effective resuscitation include over-anticipation of blood product need and poor communication between the resuscitation and laboratory teams. Collaboration between the resuscitation team and consultants in transfusion medicine is necessary for MTP protocol development to improve patient outcomes and reduce blood wastage.

## Appendices

Appendix 1: Massive Transfusion Protocol Survey

**1.     What is your current medical** **specialty** **? (select all that apply)**

ð     Emergency Medicine

ð     Anaesthesia

ð     General Surgery

ð     Critical Care/Intensive Care

ð     Hematology

ð     Hematopathology

ð     Transfusion Medicine

ð     Other (please specify)

2.     How many years have you been practicing as an attending/staff physician?

ð     0 - 5

ð     6 - 10

ð     11 - 20

ð     21 - 30

ð     > 30 years

3.     In what province do you currently spend the majority or your time practicing?  (Please specify) 

__________________________________________________________________

4.      What is the approximate population size of the city you currently practice in?  (Please specify) 

__________________________________________________________________

5.     Does the hospital in which you spend the majority of your time currently have a massive transfusion protocol (MTP)?

ð     Yes

ð     No

ð     Unsure

6.     In your view, which do you feel are appropriate activation criteria for a massive transfusion protocol? (select all that apply)

ð     > 4.5 L of blood loss in 30 minutes

ð     > 150 mL per minute of bleeding with loss of over half the circulating blood volume

ð     Peri-partum woman with marked ongoing blood loss

ð     Systolic BP < 90 and/or requiring vasopressors, from presumed haemorrhagic shock

ð     Replacement of 50% of total blood volume (TBV) within 3 hours foreseen

ð     Replacement of one entire blood volume within 24 hours

ð     Transfusion of > 4 units of packed red blood cells (pRBCs) in 1 hour when the ongoing need is foreseeable

ð     Transfusion of > 10 units of pRBCs in 24 hours foreseen

ð     Transfusion of > 20 units of pRBCs in 24 hours foreseen

ð     Other (please specify) 

7.     Of the following options, which correspond to your hospital’s/health authority’s criteria for activation of a massive transfusion protocol? (select all that apply).

ð     We do not have an MTP

ð     Don’t know

ð     > 4.5 L of blood loss in 30 minutes

ð     > 150 mL per minute of bleeding with loss of over half the circulating blood volume

ð     Peri-partum woman with marked ongoing blood loss

ð     Systolic BP < 90 and/or requiring vasopressors, from presumed haemorrhagic shock

ð     Replacement of 50% of total blood volume (TBV) within 3 hours

ð     Replacement of one entire blood volume within 24 hours

ð     Transfusion of > 4 units of pRBCs in 1 hour when ongoing need is foreseeable

ð     Transfusion of > 10 units of pRBCs in 24 hours foreseen

ð     Transfusion of > 20 units of pRBCs in 24 hours foreseen

ð     Other (please specify) 

__________________________________________________________________

8.     Considering that, on average, 1-2 units (3-15%) of all blood components are wasted per MTP activation, what do you feel is an acceptable rate of failure to activate an MTP when, retrospectively, it was appropriate to activate one (i.e, UNDER-CALL RATE)?

ð     < 5%

ð     5 - 10%

ð     11 - 20%

ð     21 - 30%

ð     > 30%

9.     If 0 units of blood components were wasted per MTP activation, what do you feel would be an acceptable UNDER-CALL RATE?

ð     < 5%

ð     5 - 10%

ð     11 - 20%

ð     21 - 30%

ð     > 30%

10.  If 4 units of blood components were wasted per MTP activation, what do you feel would be an acceptable UNDER-CALL RATE?

ð     < 5%

ð     5 - 10%

ð     11 - 20%

ð     21 - 30%

ð     > 30%

11.  Considering that, on average, 1 - 2 units (3% - 15%) of all blood components are wasted per MTP activation, what do you feel is an acceptable rate of activating an MTP when retrospectively it was inappropriate to activate one (i.e., OVER-CALL RATE)?

ð     < 5%

ð     5 - 10%

ð     11 - 20%

ð     21 - 30%

ð     > 30%

**12.  If 0 units of blood** **components were** **wasted per MTP activation, what do you feel would be an acceptable OVER-CALL RATE?**

ð     < 5%

ð     5 - 10%

ð     11 - 20%

ð     21 - 30%

ð     > 30%

13.  If 4 units of blood components were wasted per MTP activation, what do you feel would be an acceptable OVER-CALL RATE?

ð     < 5%

ð     5 - 10%

ð     11 - 20%

ð     21 - 30%

ð     > 30%

14.   Based on your experience with massive transfusion protocols and their activation, what do you feel are reasons for the current amount (3% - 15%) of blood product wasted per MTP activation? (check all that apply)

ð     Inappropriate activation of an MTP

ð     Poor communication between treating services

ð     Duration of time blood components can be out of the lab, according to locally validated MTP box time frames 

ð     Over-anticipation of the amount of blood products needed

ð     Other (please specify) 

15.  What is the maximum amount of time at your hospital that blood components can be out of the lab before they can no longer be accepted back into inventory? 

ð     1 hour

ð     4 - 7 hours

ð     8 - 12 hours

ð     > 12 hours

ð     I don’t know

16.  A 29-year-old M presents to the ED after being crushed in a mining accident. He was intubated pre-hospital and is receiving active CPR upon arrival to the ED. After inserting bilateral chest tubes, giving two 2 L of IV fluid, binding his pelvis, and placing a tourniquet on a bleeding groin injury, he obtains the return of spontaneous circulation. 

·     Vital signs: HR = 140, BP = 70/40

·     Blood products and IVF received: 2 L of Ringer’s Lactate

·     Summary of injuries: Left pneumothorax, open vertical shear pelvic fracture, vascular injury to the left iliofemoral vasculature 

·     FAST scan: Indeterminate

Based on the above would you activate an MTP?

ð     Yes

ð     No

**17.  An 80-year-old M with a PMHx of** **HTN,** **is involved in** **a MVA** **and partially ejected from his vehicle. Pre-hospital vitals revealed** **a HR** **of 110, a palpable BP of 60, with an obvious deformity to his right hip and a possible pelvic fracture.**

·     Vital signs: HR = 110, BP = 65/40

·     Blood products and IVF received: 1L of Ringer’s Lactate

·     Summary of injuries: Large forehead laceration. Possible traumatic brain injury. Right Femoral Neck Fracture, Grade 2 anterior compression fracture of the pelvis 

·     FAST scan: Indeterminate

Based on the above would you activate an MTP?

ð     Yes

ð     No

18.  A 69-year-old M, POD #0 for a ruptured AAA repair. He is intubated and ventilated in the ICU and develops hypotension. 

·     Vital signs: HR = 140, BP = 78/45

·     Blood products and IVF received: 6 L of Ringer’s Lactate (2 L in ICU), 4 U of blood, 1 L of FFP, 6 U of platelets during the initial OR

·     Summary of injuries: Recent ruptured AAA

·     FAST scan: Positive

Based on the above would you activate an MTP?

ð     Yes

ð     No

19.  A 27-year-old F, G1P0 at 28 weeks gestational age, presents to the ED with severe abdominal pain, and vaginal bleeding. 

·     Vital signs: HR = 150, BP=86/45

·     Blood products and IVF received: 3 L of Ringer’s lactate, 4 U of pRBCs 

·     Summary of injuries: Presumed placental abruption

·     FAST scan: No free fluid. Large anechoic intrauterine stripe

Based on the above would you activate an MTP?

ð     Yes

ð     No

**20.  A** **72-year-old M,** **presents to the ED after sustaining a PEA arrest *2. After CPR he achieves a return of spontaneous circulation. He is intubated and started on a norepinephrine infusion. Post-resuscitation EKG shows a new LBBB. He is being treated by Cardiology as a** **STEMI** **and given heparin with a plan to go to the Cath lab. While waiting for Cath lab, his Hbg comes back at 41. The patient is subsequently found to have maroon hematochezia on rectal exam. **

·     Vital signs: HR = 120, BP = 80/50 (on 0.3 mcg/kg/min of norepinephrine)

·     Blood products and IVF received: 3 L of Ringer’s lactate, 50 mg of protamine sulfate, 2 U of PRBCs

·     Summary of injuries: Presumed upper GI bleed

·     FAST scan: No pericardial effusion, no pleural effusion, no free fluid.

Based on the above would you activate an MTP?

ð     Yes

ð     No

21.  If possible, would you please consider providing a few names and contact e-mails of specialists working in the above-mentioned specialties.

ð     Please specify:

____________________________________________________________

Acronyms: AAA: abdominal aortic aneurysm; BP: blood pressure; CPR: cardiopulmonary resuscitation; ED: emergency department; EKG: electrocardiogram, FAST: Focused Assessment with Sonography for Trauma; F: female; FFP: fresh frozen plasma; GI: gastrointestinal; Hgb: hemoglobin; HR: heart rate; HTN: hypertension; ICU: Intensive Care Unit; IVF: intravenous fluid; L: liters; LBBB: left bundle branch block, M: male; MTP: Massive Transfusion Protocol; MVA: motor vehicle accident; OR: operating room; PEA: pulseless electrical activity; PMHx: previous medical history; POD: postoperative day; pRBCs: packed red blood cells; STEMI: ST elevation myocardial infarction; TBV: total blood volume; U: units
